# A survey of gastrointestinal helminth infestation in smallholder backyard pigs and the first molecular identification of the two zoonotic helminths *Ascaris suum* and *Trichuris suis* in Myanmar

**DOI:** 10.1186/s12917-024-03998-w

**Published:** 2024-04-06

**Authors:** Saw Bawm, Lat Lat Htun, Hla Myet Chel, Yadanar Khaing, Myint Myint Hmoon, Su Su Thein, Shwe Yee Win, Nyein Chan Soe, Yu Nandi Thaw, Naoki Hayashi, Mar Mar Win, Nariaki Nonaka, Ken Katakura, Ryo Nakao

**Affiliations:** 1 Department of Pharmacology and Parasitology, University of Veterinary Science, Yezin, Nay Pyi Taw, 15013 Myanmar; 2 Department of Livestock and Aquaculture Research, Yezin, Nay Pyi Taw, 15013 Myanmar; 3 Department of International Relations and Information Technology, University of Veterinary Science, Yezin, Nay Pyi Taw, 15013 Myanmar; 4 Laboratory of Parasitology, Graduate School of Infectious Diseases, Faculty of Veterinary Medicine, Hokkaido University, Sapporo, 060-0818 Japan; 5 Rector Office, University of Veterinary Science, Yezin, Nay Pyi Taw 15013, Myanmar

**Keywords:** *Ascaris suum*, *Trichuris suis*, Backyard pigs, Helminth parasites, Myanmar

## Abstract

**Background:**

Parasitic infestations have a substantial economic impact on pig production. This study aimed to investigate the gastrointestinal (GI) helminths in pigs and to molecularly characterise two important nematodes, *Ascaris* and *Trichuris* species.

**Materials and methods:**

A total of 500 pig faecal samples were collected from small holder backyard pig farms in five townships within Nay Pyi Taw, Myanmar. Microscopic examination was conducted to estimate the prevalence of GI helminth infestation in the pigs. DNA extraction and PCR were performed on faecal samples that were morphologically positive for *Ascaris* and *Trichuris* eggs. Molecular analysis was then conducted to characterise *A. suum* and *T. suis*, the most common and zoonotic helminths.

**Results:**

According to microscopic examination, 69.2% (346/500) were positive for GI helminth eggs. The GI helminth species observed were *A. suum*, Strongyle, *Strongyloides* spp., *T. suis*, *Metastrongylus* spp., *Hyostrongylus* spp., *Fasciolopsis* spp., *Paragonimus* spp., and *Schistosoma* spp., with occurrences of 34.8%, 29.6%, 21.4%, 20.0%, 4.0%, 1.6%, 1.0%, 1.0%, and 0.4%, respectively. Mixed infections of GI helminths were noted in 31.0% of the samples. Overall, sampled pigs excreted mostly low levels (< 100 EPG) or moderate levels (> 100–500 EPG) of GI helminth eggs. The highest mean EPG for each parasite species was noted in *A. suum*. The presence of *A. suum* and *T. suis* was confirmed molecularly. The sequences of the internal transcribed spacer 1 (ITS1) region of *A. suum* showed high similarity with previously reported sequences. Likewise, the sequences of *T. suis* exhibited high similarity with the sequences reported from humans and pigs. Age was noted as an associated factor (*P* < 0.05) for GI helminth infection status.

**Conclusions:**

In this report, *A. suum* and *T. suis* were molecularly identified for the first time in Myanmar. It is important to extend the information among the farmers to be aware of the necessity of preventing zoonotic parasites by practicing regular deworming, proper use of anthelmintics and maintaining hygienic conditions in their pig farms.

**Supplementary Information:**

The online version contains supplementary material available at 10.1186/s12917-024-03998-w.

## Background

Pig productivity is under threat due to a wide range of pig diseases. Infectious diseases have a substantial economic impact on pig production because they can reduce productivity and reproduction, as well as increase morbidity and mortality [1]. Parasites are frequently reported as the cause of diseases in pigs, particularly in tropical regions. Among parasitic diseases, gastrointestinal (GI) parasites are responsible for substantial loss of productivity in pigs in terms of inefficient feed conversion, poor growth rate, intestinal malabsorption, reduced weight gain, decreased litter size delayed or incomplete immunity subsequent to vaccinations, negative effects on meat quality and the condemnation of affected organs after slaughter [2]. Additionally, several swine parasites can be transmitted from pigs to humans, posing a significant hazard for the producer [3].

The most common helminth infestations in humans and pigs around the world are caused by nematode worms of the genera *Ascaris* and *Trichuris* [4]. Some parasites, such as *Ascaris suum* and *Trichuris suis*, are widespread in pigs and can infect both humans and pigs [4, 5]. The worms that infect pigs and humans are morphologically similar and difficult to distinguish due to a lack of distinct characteristics [5]. Infection with *A. suum* may result in production losses due to altered carcass composition, lower weight gain, and liver condemnation [6]. Furthermore, the level of natural worm cross-transmission between pig and human hosts is unknown; nevertheless, experimental cross-infections have proven that *A. suum* can infect humans, and human zoonotic cases have been reported [5, 7, 8]. Infection with *T. suis* (whipworm) in pigs can result in anorexia and bloody diarrhoea in growing pigs, which can lead to economic losses [9]. Because of the similarities among *Trichuris* species, morphological differentiation is very difficult. Moreover, the degree of natural *Trichuris* cross-transmission between people and pigs is unknown [5]. Although adult worms rarely persist, investigations on experimental infection have shown that human whipworm *T. trichiura* can establish in pigs, whereas patent *T. suis* infection has been noted in humans [5]. Because species discrimination by egg morphology is challenging, the development of molecular approaches for species identification and diversity evaluation is extremely beneficial [10]. Several molecular markers have been used for the identification of *Ascaris* and *Trichuris* spp., such as nucleotide sequences of internal transcribed spacers 1 and 2 (ITS1 and ITS2) [11, 12].

The pig population in Myanmar in 2021 was estimated to be approximately 6.8 million [13]. Approximately 86% of the Myanmar population resides in rural areas and is involved in agro-livestock production and agro-industrial work. Most pig farmers in Myanmar prefer backyard farming due to its simplicity and low cost, generally with intensive or semi-intense methods [14]. Even though pig production in Myanmar contributes to farmers maintaining a sustainable livelihood, there are many challenges to maintaining productivity, profitability and sustainability. One of these challenges is parasitic infestation. Although our recent study demonstrated the occurrence of *Cystoisospora* infection in pigs in Myanmar [15], data on helminth infestation are very limited. Therefore, this study aimed to investigate the GI helminths in pigs and to molecularly characterize two important nematodes, *Ascaris* and *Trichuris* species, in the Nay Pyi Taw area, Myanmar.

## Results

### Parasite detection rate

**Fig. 1:**
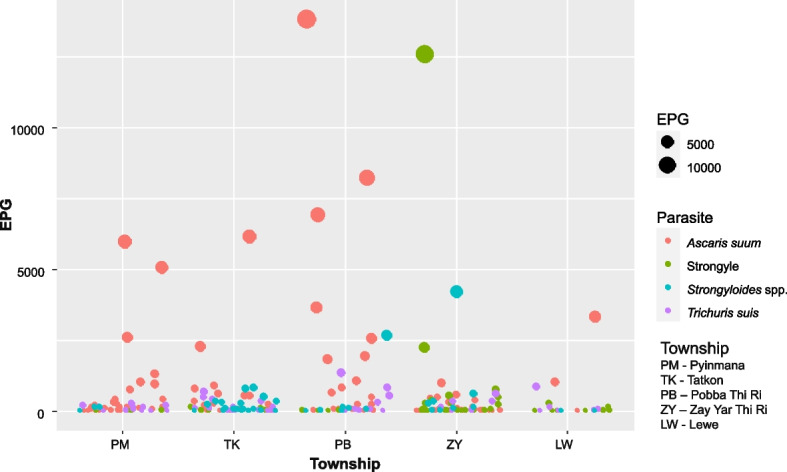
EPG of the most common GI helminth species (*Ascaris* sp., Strongyle, *Trichuris* sp. and *Strongyloides* spp.) observed in five townships

The overall occurrence of GI helminth infestation among pigs was 69.2% (346/500). The helminth species observed were *A. suum*, Strongyle, *Strongyloides* spp., *T. suis, Metastrongylus* spp., *Hyostrongylus* spp., *Fasciolopsis* spp., *Paragonimus* spp., and *Schistosoma* spp. (Fig. S1 and Table 1). Overall, 31.0% of examined pigs were found to be infested with more than one GI helminth species. The occurrence of GI helminths was found to be the lowest (52.0%) in Lewe township and the highest (85.0%) in Pyinmana township. The mean eggs per gram of faeces (EPGs) for *A. suum*, *Strongyle* spp., *Strongyloides* spp. and *T. suis* were 172.7, 40.4, 27.8 and 20.5, respectively, and the mean EPGs of overall helminth species were 307.0, 214.4, 225.9, 496.7, and 63.0 in Zay Yar Thi Ri, Tatkon, Pyinmana, Pobba Thi Ri and Lewe Townships, respectively (Fig. 1).

**Table 1:** Occurrence of GI helminth infestation in pigs in Nay Pyi Taw area (*n* = 500)

No		Occurrence %	Total positive number
**Parasite species**			
1	*Ascaris* spp.	34.8	174
2	*Strongyle* spp.	29.6	148
3	*Strongyloides* spp.	21.4	107
4	*Trichuris* spp.	20.0	100
5	*Metastrongylus* spp.	4.0	20
6	*Hyostrongylus* spp.	1.6	8
7	*Fasciolopsis* spp.	1.0	5
8	*Paragonimus* spp.	1.0	5
9	*Schistosoma* spp.	0.4	2
10	*Macracanthorhynchus* spp.	0.2	1
**Distribution among township**			
1	Pyinmana	85	85
2	Tatkon	77	77
3	Pobba Thi Ri	76	76
4	Zay Yar Thi Ri	56	56
5	Lewe	52	52
	Overall	69.2	346

### Distribution of GI helminth infestation among age groups, feeding systems, animal housing floors, hygiene conditions and associated factors

**Fig. 2:**
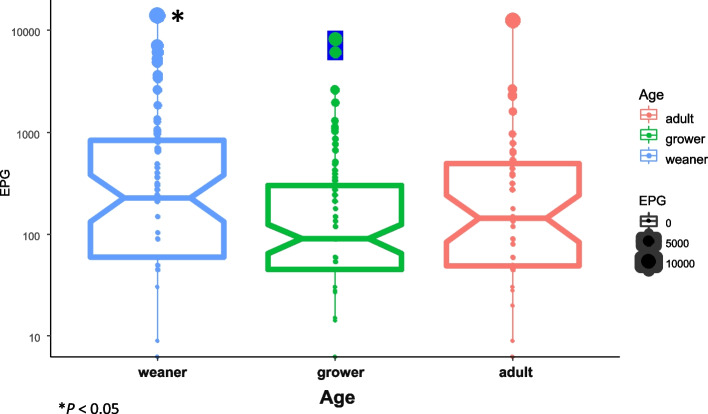
The intensity of helminth infestation (EPG) was higher (*P* < 0.05) in weaners than in growers and adults. The top and bottom horizontal lines of the boxplots represent the first and third quartiles of the data range, respectively. The medians are shown by middle horizontal lines, and the data range is shown by vertical lines, with outliers plotted as points. The notches of each boxplot are approximate 95% confidence intervals of medians

The mean EPG was 261.4 (ranging from 16.7 to 13,800) for overall helminth infestation in this study. The mean EPG (Mean ± SD) values in age groups were 1031 ± 2,023 (Median = 225) in weaners, 429 ± 1177 (Median = 90) in growers and 646 ± 1862 (Median = 143) in adults. Among the age groups, the weaner group showed the highest EPG when compared to the other age groups (*P* < 0.05) (Fig. 2). Although not significantly different, the highest intensity of helminth infection was found in pigs fed local feed (827 ± 1,946 EPG and median = 150), followed by pigs fed mixed feed (573 ± 1640 EPG and median = 1,640) and pigs fed commercial feed (306 ± 264 EPG and median = 330). In this study, a higher intensity of helminth infestation was found in farms with ground floors (799 ± 1,525 EPG and median = 150), and a lower intensity was found in farms with concrete floors (643 ± 1,836 EPG and median = 120). Furthermore, a higher intensity of helminth infestation was found in farms with no hygiene practices (799 ± 1,525 EPG and median = 150) than in farms with hygiene practices (643 ± 1,836 EPG and median = 120) (Fig. S2). According to the chi-square test, age was identified as the only factor associated (*P* < 0.05) with the occurrence of GI helminth infestations in pigs in this study (*P* = 0.026, χ^2^ = 7.306). Breed, anthelmintic treatment, floor type, feed type and hygienic condition of the farm were not associated with the occurrence of GI helminth infestations.

### Molecular identification of *Ascaris suum* and *Trichuris suis*

**Fig. 3:**
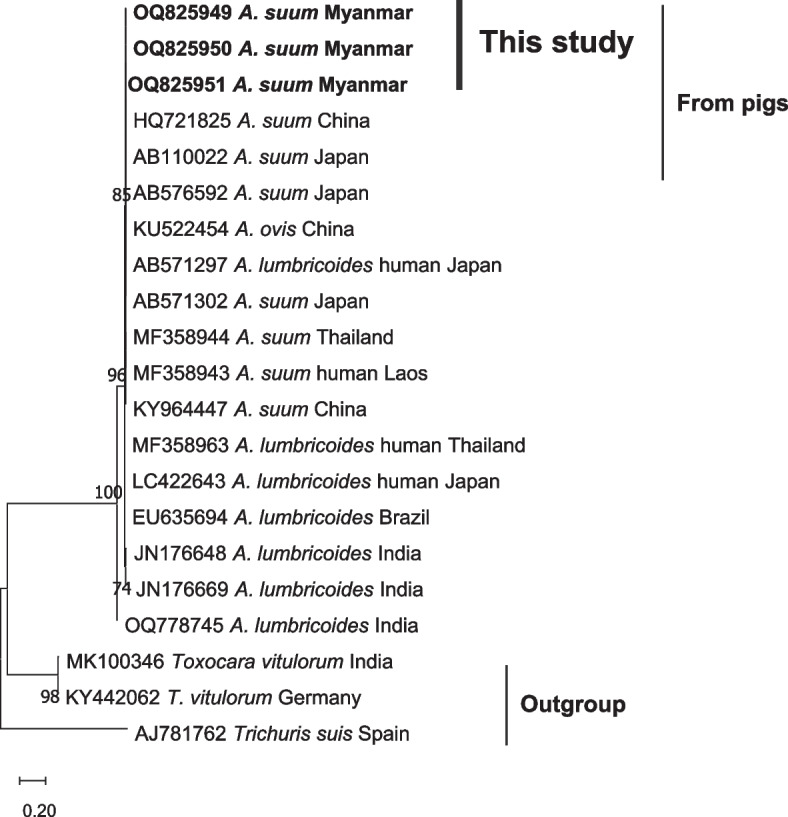
The phylogenetic relationship of partial ITS1 sequences of *Ascaris suum* detected in this study and reference sequences. The phylogenetic tree was constructed by the maximum likelihood method based on the Tamura-Nei model. The bold taxa represent the sequences obtained from the current study. The GenBank accession number of each sequence is given. Bootstrap values were computed independently for the purposes of 1000 replicates

**Fig. 4:**
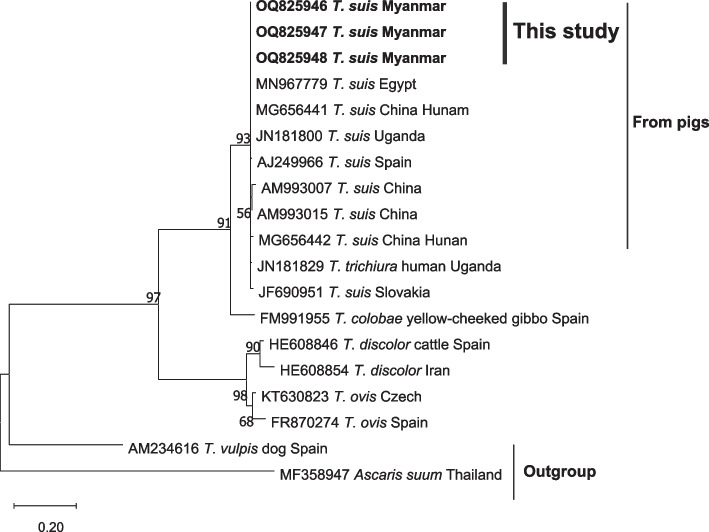
The phylogenetic relationship of partial ITS2 sequences of *Trichuris suis* detected in this study and reference sequences. The phylogenetic tree was constructed by the maximum likelihood method based on the Tamura-Nei model. The bold taxa represent the sequences obtained from the current study. The GenBank accession number of each sequence is given. Bootstrap values were computed independently for the purposes of 1000 replicates

PCR was performed on 15 samples from each of the *A. suum-* and *T. suis*-positive samples by microscopic examination. Sequencing was performed on three samples with a targeted size of ~ 515 bp for the *A suum*-specific ITS1 PCR products. The obtained nucleotide sequences were all clustered together with *A. suum* sequences available in the database (Fig. 3), with being 99.6 to 100% identical to *A. suum* from pigs in Japan (AB576592, AB571302 and AB110022), China (HQ721825) and Thailand (MF358944), and one from humans in Lao PDR (MF358943). Three samples were used for sequencing of the *T. suis*-specific ITS2 PCR products with a targeted size of ~ 635 bp. The obtained nucleotide sequences were all clustered together with *T. suis* sequences available in the database (Fig. 4), showing 100% identity to the sequences of *T. suis* from pigs in Egypt (MN967779) and 99.5%, 99.1%, and 98.7% identities to *T. suis* sequences from pigs in China (MG656441, AM993015 and AM993007), Uganda (JN181800), and Spain (AJ249966), respectively (Table 2).

**Table 2:** Identification of ITS1 sequences of *Ascaris suum* or ITS2 sequences of *Trichuris suis* isolates from pigs in Myanmar using the NCBI BLAST search

**Study site**	**Sample ID**	**Length (bp)**	Closest sequences in GenBank data bases				
			**Identity (%) and remarks**	**Species (taxon name)**	**GenBank accession no**	**Host**	**Country**
**ITS1 sequences of *A. suum***							
Nay Pyi Taw	P-28	515	100% identity among isolated sequences	*Ascaris suum*	OQ825949	pig	This study
Nay Pyi Taw	P-187	515		*A. suum*	OQ825950	pig	This study
Nay Pyi Taw	P-402	515		*A. suum*	OQ825951	pig	This study
			485/485 (100%)	*A. suum*	AB576592	pig	Japan
			493/494 (99.8%)	*A. suum*	AB571302	pig	Japan
			502/504 (99.60%)	*A. suum*	MF358944	pig	Thailand
			505/507 (99.61%)	*A. suum*	MF358943	human	Lao
			486/487 (99.79%)	*A. suum*	AB110022	pig	Japan
			482/483 (99.79%)	*A. suum*	HQ721825	pig	China
			493/494 (99.8%)	*A. lumbricoides*	AB571297	human	Japan
			493/495 (99.6%)	*A. ovis*	KU522454	sheep	China
			489/494 (98.99%)	*A. lumbricoides*	LC422643	human	Japan
			507/514 (98.64%)	*A. lumbricoides*	MF358963	human	Thailand
			508/514 (98.83%)	*A. suum*	KY964447	pig	China
			391/393 (99.49%)	*A. suum*	ON493798	pig	Brazil
**ITS2 sequences of *T. suis***							
Nay Pyi Taw	P-21	635	100% identity among isolated sequences	*Trichuris suis*	OQ825946	pig	This study
Nay Pyi Taw	P-180	635		*T. suis*	OQ825947	pig	This study
Nay Pyi Taw	P-399	635		*T. suis*	OQ825948	pig	This study
			408/408 (100%)	*T. suis*	MN967779	pig	Egypt
			632/635 (99.53%)	*T. suis*	MG656441	pig	China Hunam
			529/534 (99.06%)	*T. suis*	JN181800	pig	Uganda
			529/536 (98.69%)	*T. suis*	AJ249966	pig	Spain
			527/533 (98.87%)	*T. suis*	AM993007	pig	China
			630/634 (99.37%)	*T. suis*	AM993015	pig	China
			629/640 (98.28%)	*T. suis*	MG656442	pig	China
			529/537 (98.51%)	*T. trichiura*	JN181829	human	Uganda
			599/619 (96.77%)	*T. suis*	JF690951	pig	Slovakia

## Discussion

Pigs are frequently affected by helminth parasites worldwide in all types of production systems. The findings of this study were the first to reveal the occurrence of GI helminths in pigs in Myanmar as well as the molecular identification of pig helminths. This study found a higher occurrence of 62.9% compared to 56.95% in Rajasthan district, India [16], and 37.5% in Aizawl district of Mizoram, India [17]. The differences in the infection rates were attributed to factors such as breed, geographical condition, climate, hygiene and faecal examination techniques [18]. The higher occurrence could be attributed to the fact that all of the farms enrolled in this study were smallholder farms with poor hygienic conditions. Many investigations stated that the spread of intestinal helminths in pigs raised using traditional systems was due to poor hygiene, poor nutrition and inadequate anthelmintic interventions [19, 20].

Among the helminths observed in this study, *A. suum* was the dominant species, with an occurrence of 34.8% and the highest mean EPG of 173. Although the prevalence varies greatly with the environment and production system, *A. suum* is the most or second most common intestinal species in farmed pigs globally [6). The higher prevalence of *Ascaris* sp. was also investigated in Denmark (88%) [21], the Netherlands (72.7%) [22], Nagaland, India (65.5%) [23], Vietnam (51%) [24], Nepal (45.0%) [25], South Africa (44.5%) [26], Burkina Faso (40%) [18], and China (36.7%) [27]. A lower prevalence was reported in India (11.1–33.3%) [28, 29], Kenya (28.7%) [19], and Rwanda (10.6%) [30].

According to Băieş et al. [31], *A. suum* is the most common endoparasite of swine in most countries and is one of the most economically important parasites. Thus, the highest occurrence of *Ascaris* observed in this study should be considered for worm control, although the sampled pigs were apparently healthy. Moreover, the zoonotic potential of *Ascaris* should also be considered because its occurrence (34.8%) in the present study was high. Among the ten helminth species observed, *Strongyloides* spp., *Trichuris* sp., *Fasciolopsis* spp., and *Schistosoma* spp. are zoonotic helminths with occurrences of 21.4%, 20%, 1%, and 1%, respectively. Therefore, it is crucial to manage the smallholder pig farms in the Nay Pyi Taw area to lessen the impact of helminth infestations by practicing proper deworming strategies.

Strongyle was observed as the second most prevalent nematode, with 29.6% occurrence. It was lower than the reports from Uganda, 89% [32], Kenya, 75% [33], Tanzania, 52% [34], Brazil, 46.6% [35] and Nepal, 32% [25], while lower than the findings from Ghana, 11% [36] and India, 11.10% [28]. *Strongyloides* spp. was observed in 21.4% of samples. Higher prevalence rates were reported from Bangladesh, 29.1% [20], Kenya, 26.6% [33], and Nepal, 23% [25], and lower prevalence rates were described from Indonesia, 19% [37], Tanzania, 15% [34] and India, 12.74% [38].

The 20.0% occurrence of *T. suis* noted in this study was in agreement with the report from Indonesia, 20.0% [37]. This occurrence was lower than the findings from Kenya, 78.0% [33], South Africa, 50.6% [26], the Netherlands, 37.5% [22], Nepal, 30% [25], India, 27.84% [28] and Japan, 24.8% [39] and higher than the findings from India, 17.3% [40] and Uganda, 17% [32]. The other observed helminths, *Metastrongylus* spp., *Hyostrongylus* spp., and *Paragonimus* spp. were found to have lower infection rates, with occurrence rates of 4.0%, 1.6%, and 1.0%, respectively.

According to the statistical analysis, the occurrence of GI helminth infestation was found to be associated with swine age in this study. Pigs < 3 months of age had an increased risk of infestation when compared to older pigs. The other hypothesized factors, breed, anthelmintic treatment, floor type, feed type and hygienic condition of pig farm, appeared to have no association with GI helminth infestation in this study. For the age factor in this study, a higher infestation rate was noted in younger pigs. Immunity to GI helminths might develop in older pigs and thus could be a reason for the higher occurrence in younger pigs. Foster and Elsheikha [41] also pointed out that the lower prevalence of GI parasites in adult pigs could be due to the enhanced resistance and susceptibility to reinfection governed by increased immunological memory.

In the studied townships, the GI helminths’ occurrences were different with the minimum (52.0%) in Lewe Township and maximum (85.0%) in Pyinmana Township. However, farm management practices, such as deworming, hygienic conditions, feed type and floor type, were not much different among the townships. The significant association (*P* < 0.05) could be explained by the fact that sampling in Pyinmana Township was conducted at the end of May when the rainy season starts in Myanmar. In the remaining four townships, sampling was performed in the months of December and January. Parasitic worm eggs and larval development prefer a moist environment, and thus, the parasitic contamination rate increased in pigs and the environment. In accordance with this fact, it could be considered that the highest occurrence was noted in Pyinmana Township.

Overall, sampled pigs excreted mostly low levels (< 100 EPG) and moderate levels (> 100–500 EPG) of GI helminths. Thus, it is clear that the sampled pigs seemed to be normal in clinical appearance without showing any abnormalities due to harbouring moderate levels of GI helminth infestations. However, de Araújo et al. [42] suggested that subclinical infections are important and can be frequent, resulting in loss of appetite, low weight gain, and reduced feed conversion in affected animals.

The eggs of most common worms that infect pigs and humans (*A. suum* and *T. suis*) are morphologically similar to other species of the same genera and thus the ITS1 and ITS2 sequences have frequently been used for distinguishing closely related species [43]. In this investigation, we identified and examined the sequences of the ITS1 region of *A. suum* and the ITS2 region of *T. suis* and compared them to previously published sequences of those infecting humans and pigs from various regions. Sequence analysis of *A. suum* identified from humans and pigs revealed that the isolates belong to the same clade as the sequences documented in Asian countries and Brazil. Additionally, sequences of *T. suis* identified from pigs and *T. trichiura* from humans also belong to the same clade. These findings suggest that humans and pig-derived *A. suum* exhibit genetic similarities with sequences reported from Asian countries and that *T. suis* likewise shares genetic similarities between humans and pigs. Therefore, this finding supported the findings of Sadaow et al. [44], who assumed that zoonotic cross-transmission of *Ascaris* roundworm between pigs and humans might exist in Thailand, Lao PDR, and Myanmar. Although the utilization of ITS1 and ITS2 sequences is beneficial in distinguishing between parasite species [11], their resolution power is comparatively lower than that of other genome-wide identification techniques.

## Conclusion

The overall GI parasite infestation rate in pigs was 69.2%, with 31.0% mixed infections, and five species of zoonotic helminths were found in the present study. Furthermore, age was a major factor related to GI helminth infestation in smallholder backyard pigs in the Nay Pyi Taw area. The two zoonotic helminths, *A. suum* and *T. suis*, were molecularly identified for the first time. It is important to extend the information among the farmers to be aware of the importance of prevention of zoonotic parasites for public health by practicing regular deworming, proper use of anthelmintics and keeping hygienic conditions in their pig farms.

## Methods

### Sample size and sample collection

The study area, sample size and sampling period were reported in our previous study [15]. In brief, a cross-sectional study was conducted in five townships within Nay Pyi Taw, located between latitude 19° 45′ N and longitude 96° 06′ E, between December 2020 and May 2021. Samples were collected mostly in suburban and rural areas within townships, which were home to a large number of pig farms. A total of 500 fresh faecal samples were obtained, with 100 samples from each township. Upper parts of freshly dropped faeces on the ground of individual pigs were collected, placed into individual zip lock bags, labelled, put in an ice box, and brought to the Laboratory of Department of Pharmacology and Parasitology, University of Veterinary Science. During sampling, information regarding the age, sex, breed, type of feed, floor type and hygienic condition of the pig farm, as well as anthelmintic usage, were all recorded. The pig breeds included local and DYL (a cross breed of Duroc, Yorkshire, and Landrace). According to Esrony et al. [45], animals were classified as weaners (5–12 weeks), growers (> 12 weeks to 24 weeks), and adults (> 24 weeks).

### Laboratory analysis for parasite eggs

The samples were examined for helminth parasites in the laboratory using the faecal flotation and sedimentation methods as described by Zajac and Conboy [46]. On the basis of morphological characteristics described by Taylor et al. [47], parasite species were identified. The McMaster egg counting method was used to calculate the number of eggs per gram (EPG) of faeces [46].

### DNA extraction and PCR

DNA extraction was performed on faecal samples that were positive for *A. suum* or *T. suis* eggs by fecal examination. According to the manufacturer’s instructions, DNA was extracted using the Power Fecal DNA Isolation kit (MO BIO Laboratories, USA). The extracted DNA samples were eluted in 200 μl elution buffer and kept at -80 °C. A NanoDrop 2000 spectrophotometer (Thermo Fisher Scientific, MA, USA) was used to determine the DNA concentration. To amplify the ITS1 region of *A. suum*, a primer set consisting of the forward primer F2662 (5'-GCAAAAGTCGTAACAAGGT-3') and the reverse primer R3214 (5'- CTGCAATTCGCACTATTTATCG-3') was employed [11]. For the amplification of the ITS2 region of *T. suis*, a primer set of the forward primer ITS2_tt_F2 (5'-GCTCGTAGGTCGTTGAAG-3') and the reverse primer ITS2_tt_R2_new1 (5'-GGGCAGCTTCCGTACT-3') was used [12]. Thermal cycling began with denaturation at 94 °C for 1 min, then 40 cycles at 98 °C for 10 s, 52 °C (for *A. suum*) and 54 °C (for *T. suis*) for 15 s, 68 °C for 1 min, and a final extension at 68 °C for 5 min. DNA samples from previously collected *A. suum* and *T. suis* specimens were used as positive controls, with molecular grade deionised water serving as negative controls. PCR products were examined using 2% Tris–acetate-EDTA (TAE) agarose gel electrophoresis after staining with RedSafe Nucleic Acid Staining Solution (iNtRON Biotechnology Inc., Seongnam, Korea).

### Sequencing and phylogenetic analysis

Following the manufacturer's instructions, DNA fragments obtained from the PCR were excised from the gel and purified using a NucleoSpin® Gel and PCR Clean-up Kit (MACHEREY–NAGEL, Düren, Germany) and submitted for direct sequencing on an Applied Biosystems 3130 Genetic Analyzer with a BigDye v3.1 Terminator cycle sequencing kit (Applied Biosystems, Inc., Carlsbad, CA, USA). ATGC version 7 (GENETYX Corporation, Tokyo, Japan) was used for multiple sequence alignment. The phylogenetic analysis was conducted using the maximum likelihood (ML) method in MEGA X with Tamura-Nei model [48]. The bootstrap analysis was performed with 1000 replicates per tree. The obtained sequences (OQ825946-OQ825951) were compared to those in the NCBI nucleotide database (http://www.ncbi.nlm.nih.gov/nuccore/).

### Statistical analysis

All data were entered into Microsoft Excel 2013 and analysed using the Statistical Package for Social Science (SPSS) version 20.0. The association between the occurrence of GI helminths and the hypothesized factors of age, sex, breed of pigs, anthelmintic treatment, floor type, feed type, and hygienic condition of the pig farm was analysed using a Pearson’s Chi-square test at the *P* < 0.05 level of significance. The boxplots were explored by the ggplot package using the R language platform [49, 50].

### Supplementary Information

**Additional file 1: Fig. S1. **(A-I). Eggs of *Ascaris suum *(A), *Oesophagostomum *spp. (B), *Strongyloides *spp. (C), *Trichuris suis* (D), *Metastrongylus* spp. (E), *Hyostrongylus* spp. (F), *Fasciolopsis* spp. (G), *Paragonimus* spp. (H), and *Schistosoma* spp. (I) detected in this study.

**Additional file 2:** **Fig. S2.** (A), (B) and (C). Intensity of helminth infestation (EPG) was lower in pigs fed with commercial feed than local and mixed feed (A), higher in pigs reared on ground floor (B) and farms with no hygienic practices (C). The top and bottom horizontal lines of the boxplots represent the first and third quartiles of the data range, respectively, the medians are shown by middle horizontal lines, and the data range is shown by vertical lines, with outliers plotted as points. The notches of each boxplot are approximate 95% confidence intervals of medians.

## Data Availability

No datasets were generated or analysed during the current study.
