# Supplementary material for: A survey of gastrointestinal helminth infestation in smallholder backyard pigs and the first molecular identification of the two zoonotic helminths Ascaris suum and Trichuris suis in Myanmar
Source: BMC Vet Res. 2024 Apr 6;20:139. doi: 10.1186/s12917-024-03998-w (PMC10998307; doi:10.1186/s12917-024-03998-w)
Supplement: Supplementary file 1 — Additional file 1: Fig. S1. (A-I). Eggs of Ascaris suum (A), Oesophagostomum spp. (B), Strongyloides spp. (C), Trichuris suis (D), Metastrongylus spp. (E), Hyostrongylus spp. (F), Fasciolopsis spp. (G), Paragonimus spp. (H), and Schistosoma spp. (I) detected in this study. [file 12917_2024_3998_MOESM1_ESM.pptx]

## Slide 1
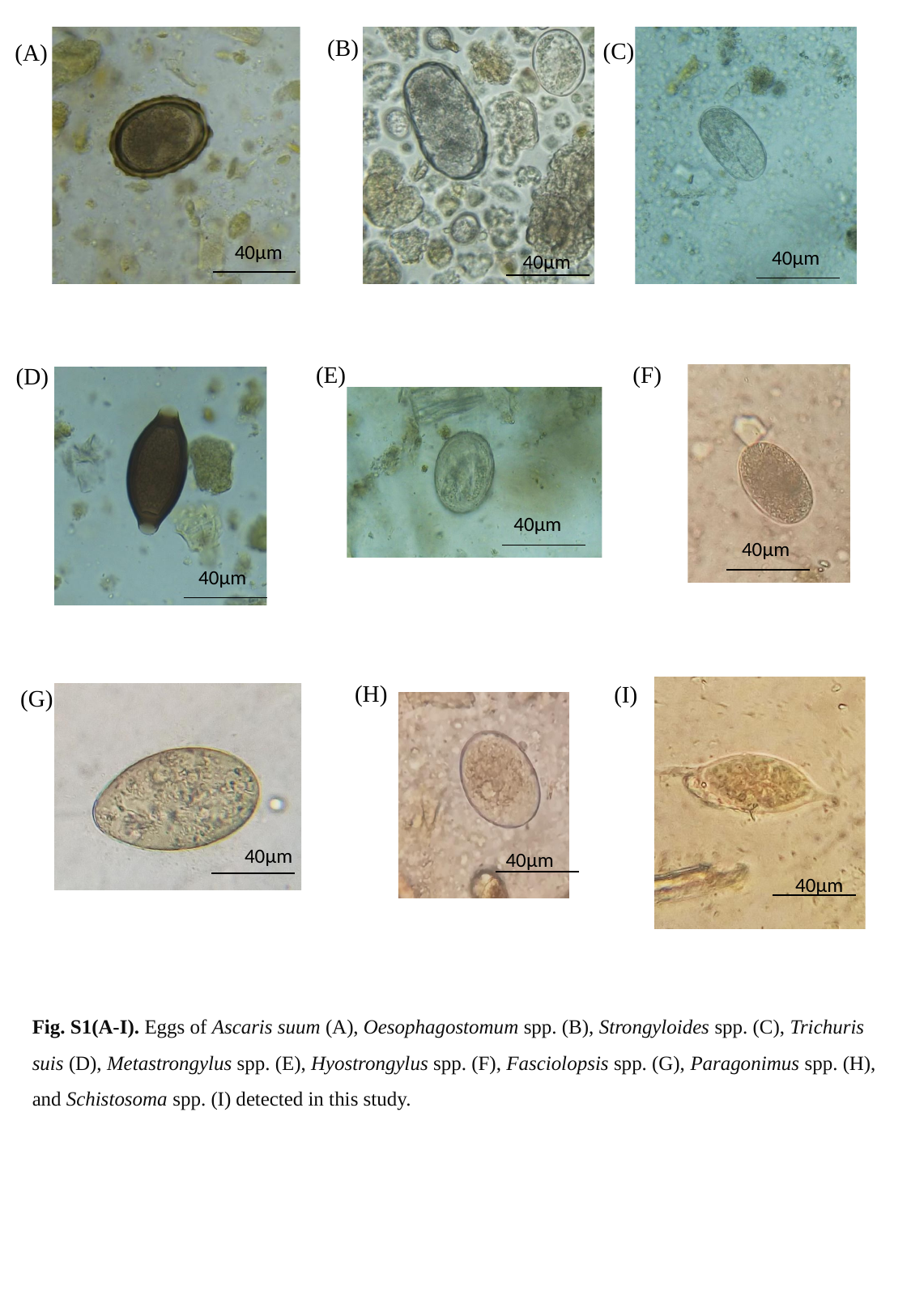

(B)
40µm
40µm
40µm
40µm
40µm
40µm
40µm
40µm
40µm
(C)
(A)
(E)
(F)
(D)
(H)
(I)
(G)
Fig. S1(A-I). Eggs of Ascaris suum (A), Oesophagostomum spp. (B), Strongyloides spp. (C), Trichuris suis (D), Metastrongylus spp. (E), Hyostrongylus spp. (F), Fasciolopsis spp. (G), Paragonimus spp. (H), and Schistosoma spp. (I) detected in this study.
