# Supplementary material for: A survey of gastrointestinal helminth infestation in smallholder backyard pigs and the first molecular identification of the two zoonotic helminths Ascaris suum and Trichuris suis in Myanmar
Source: BMC Vet Res. 2024 Apr 6;20:139. doi: 10.1186/s12917-024-03998-w (PMC10998307; doi:10.1186/s12917-024-03998-w)
Supplement: Supplementary file 2 — Additional file 2: Fig. S2. (A), (B) and (C). Intensity of helminth infestation (EPG) was lower in pigs fed with commercial feed than local and mixed feed (A), higher in pigs reared on ground floor (B) and farms with no hygienic practices (C). The top and bottom horizontal lines of the boxplots represent the first and third quartiles of the data range, respectively, the medians are shown by middle horizontal lines, and the data range is shown by vertical lines, with outliers plotted as points. The notches of each boxplot are approximate 95% confidence intervals of medians. [file 12917_2024_3998_MOESM2_ESM.pptx]

## Slide 1
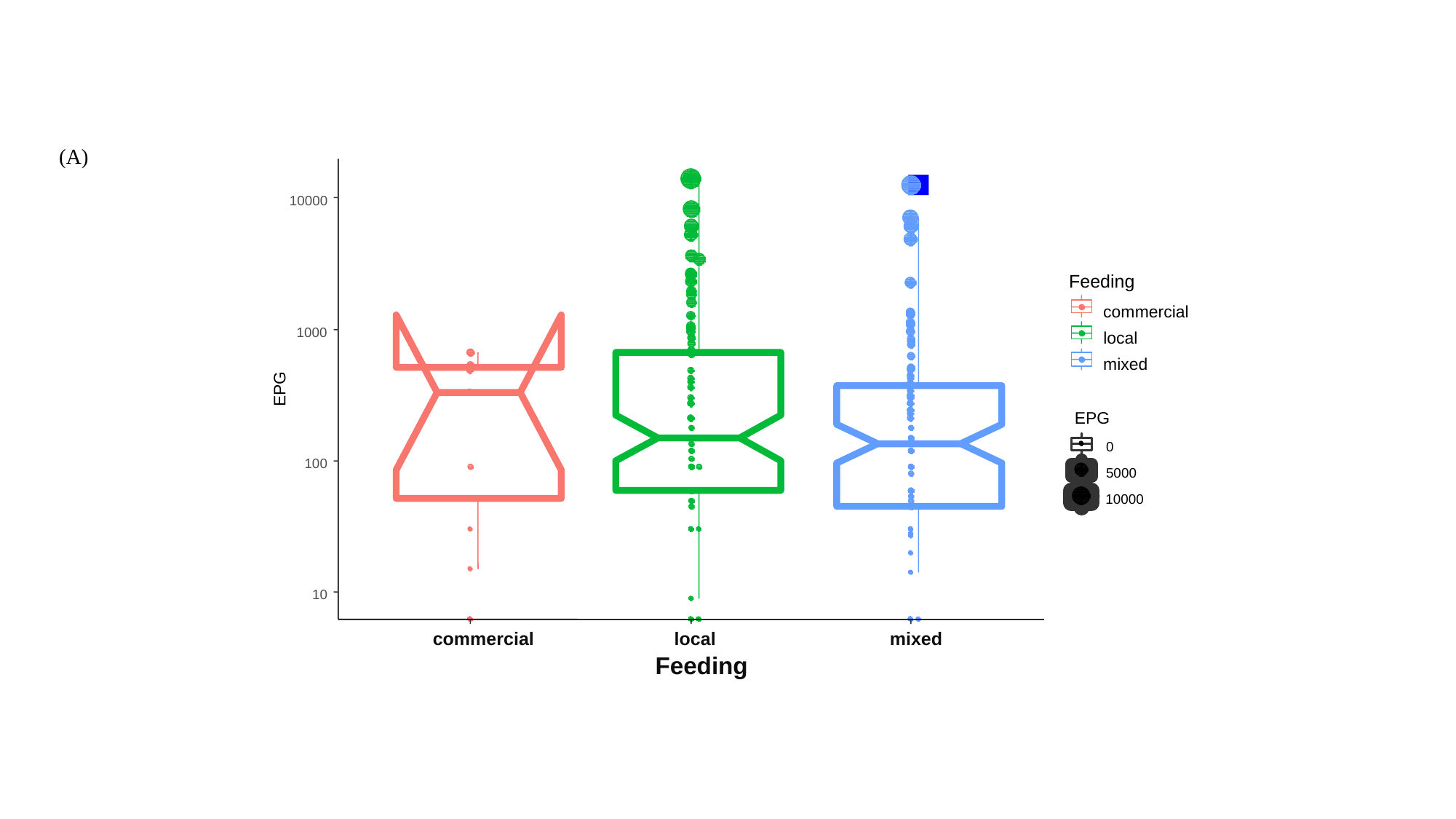

(A)
10000
Feeding
commercial
1000
local
mixed
EPG
EPG
100
10
commercial
local
mixed
Feeding
0
5000
10000

## Slide 2
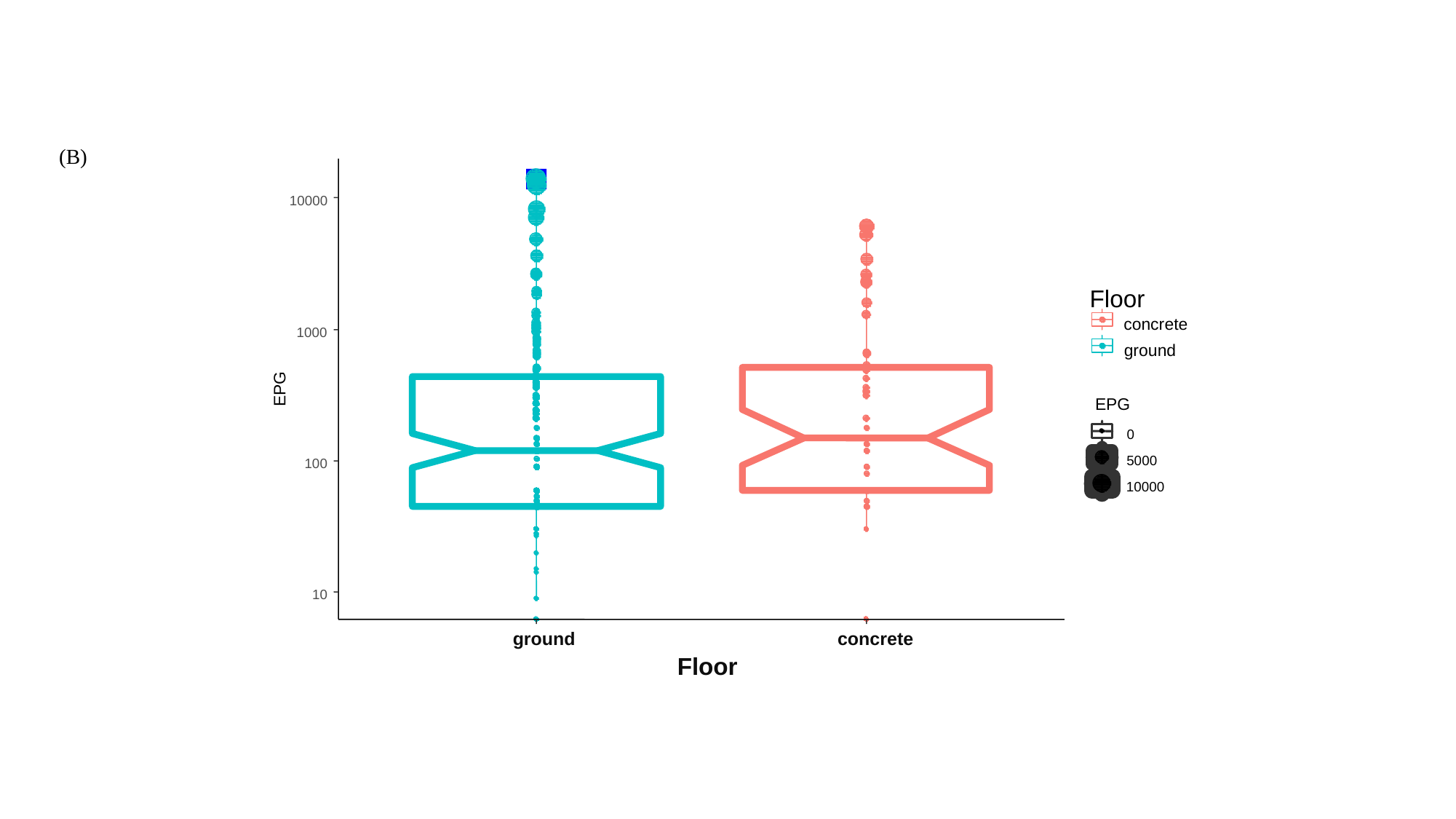

(B)
10000
Floor
concrete
1000
ground
EPG
EPG
100
10
ground
concrete
Floor
0
5000
10000

## Slide 3
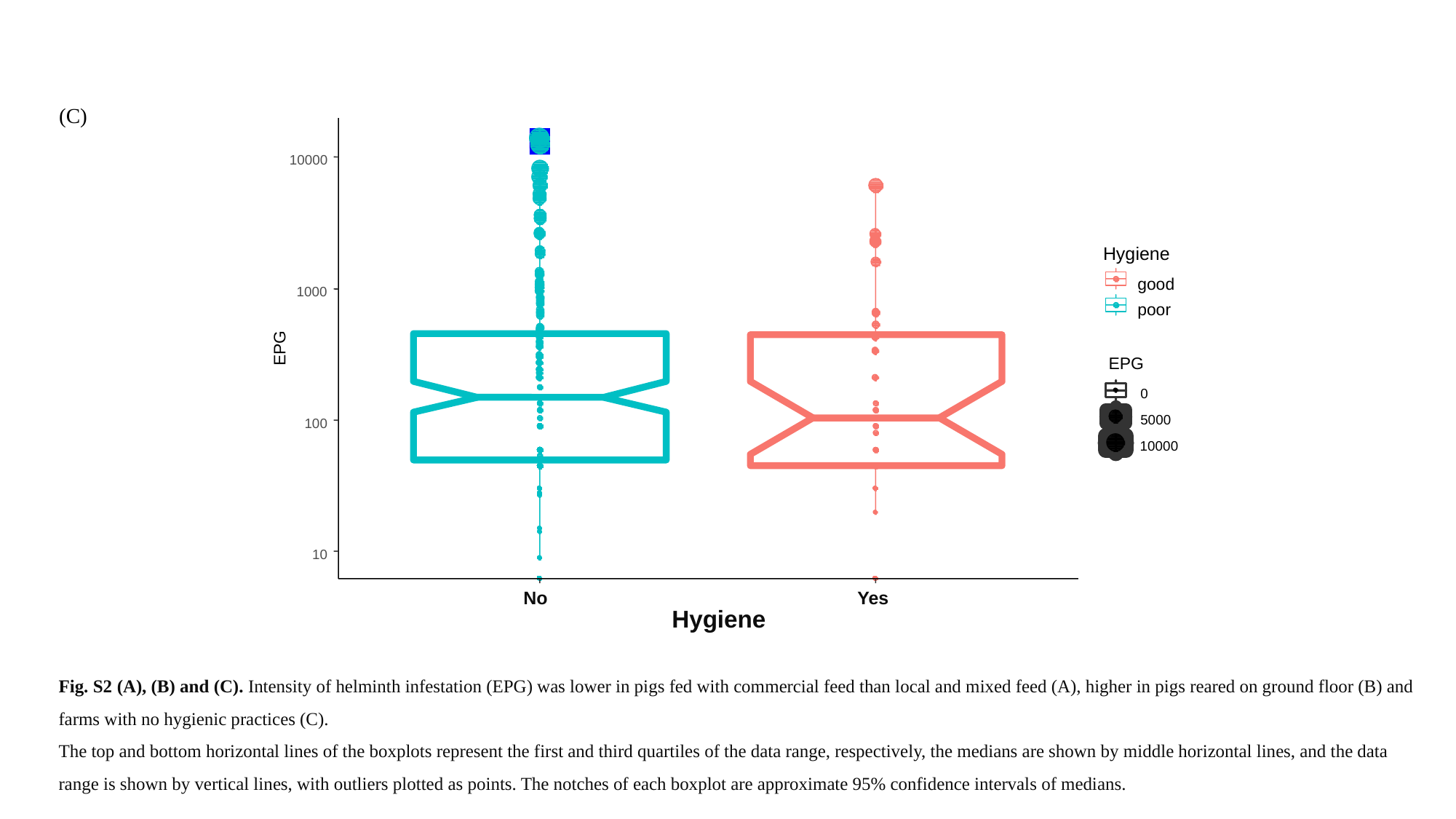

(C)
10000
Hygiene
good
1000
poor
EPG
EPG
100
10
No
Yes
Hygiene
0
5000
10000
Fig. S2 (A), (B) and (C). Intensity of helminth infestation (EPG) was lower in pigs fed with commercial feed than local and mixed feed (A), higher in pigs reared on ground floor (B) and farms with no hygienic practices (C).
The top and bottom horizontal lines of the boxplots represent the first and third quartiles of the data range, respectively, the medians are shown by middle horizontal lines, and the data range is shown by vertical lines, with outliers plotted as points. The notches of each boxplot are approximate 95% confidence intervals of medians.
